# Exploring bioadhesion: insight on innovative strategies to investigate bioadhesive scaffolds

**DOI:** 10.1016/j.ijpx.2025.100359

**Published:** 2025-07-15

**Authors:** Marta Pollini, Eleonora Bianchi, Marco Ruggeri, Barbara Vigani, Silvia Rossi, Giuseppina Sandri

**Affiliations:** Department of Drug Sciences, University of Pavia, Via Taramelli 12, 27100 Pavia, Italy

**Keywords:** Bioadhesive materials, Biomaterials, Bioadhesion, Mucoadhesion, Tissue engineering, Wound healing

## Abstract

In the pharmaceutic field, materials with enhanced bioadhesive properties have been widely employed to produce scaffolds with deep interaction and adhesion to the biological surfaces, preventing them from dislocation and promoting cell homing, proliferation and growth. Parallelly, mucoadhesion has been extensively used to increase formulation retention onto the mucosal surface. This review aims to describe the most appropriate and relevant techniques to evaluate scaffolds bioadhesion and mucoadhesion for biomedical application, and more in details, in wound healing treatment. Different methods will be reviewed and described in order to provide an overview of the traditional approaches and the most innovative and recent tools. In addition, critical considerations on the variety of biological substrates that could be used will be reported to underline the different alternatives for testing bioadhesion, including *ex-vivo* and artificial options. Biomaterials, with a particular focus on bioadhesives, will be presented, as well as the mechanisms that govern bioadhesion and mucoadhesion.

## Graphical abstract

**Figure:**
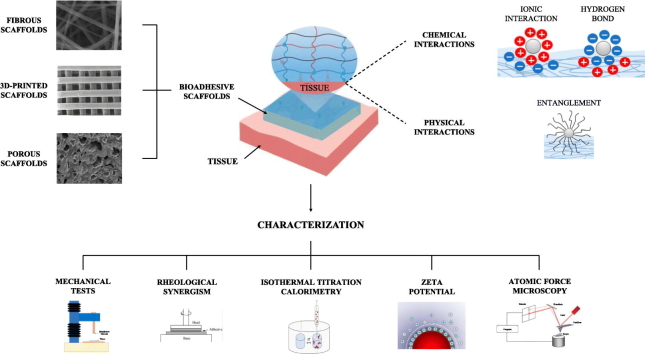

## 1 Introduction

**Fig. 1:**
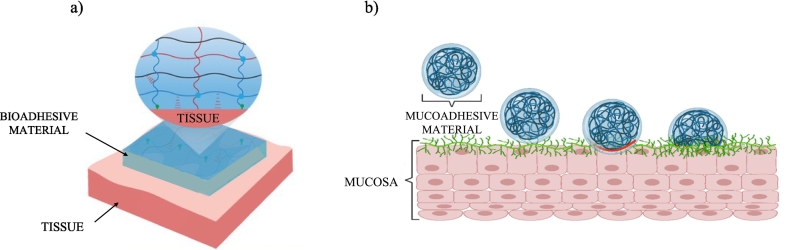
Type of interaction between materials and a) tissue (dapted with permission from (Zhang et al., 2022)); b) mucosa (dapted from (de Lima et al., 2022) CC BY 4.0).

For the first time in 1968, Baier et al. applied the concept of adhesion to the field of bioadhesion (Zhu et al., 2018). Adhesion is referred to the interaction occurred between a pressure-sensitive adhesive and a surface involving interfacial forces, including chemical and physical forces (Asati et al., 2025). In Fig. 1 the type of interaction between bioadhesioves and the biological substrates An adhesive material is a bioadhesive (a biological or a synthetis material) when it is able to interact with a biological surface, such as a cell, a tissue, or an organ (Tangri and Madhav, 2011). The process of bioadhesion causes the adhesion to a biological substrate over an extended time by means of the formation of hydrogen or ionic bonds or physical entanglements (Zhu et al., 2018; Asati et al., 2025). Furthermore, the concept of bioadhesion also includes the use of bioadhesive materials to attach two biological surfaces together (Tarafder et al., 2020). On the other hand, when a bioadhesive attaches to a mucus layer, the term used to describe the process is preferably mucoadhesion (Saha et al., 2020). In particular, due to their ability to create bonds with the mucosal layers, mucoadhesives have started to be largely employed in drug delivery to prolong pharmaceutical administration by means of local application in mucosal sites, as vaginal, buccal, nasal and ocular mucosae (Gad et al., 2023).

Over the last decades, bioadhesive and mucoadhesive materials have been intensively employed because of their multiple advantages, crucial for the formulations design and development aiming at different purposes, in both the field of drug delivery systems and medical devices. Among these, tissue sealants, tissue engineering scaffolds for wound healing and for degenerative bones treatment have been extensively studied (Zhu et al., 2018; Yadav et al., 2024; Sharma et al., 2024; Wiltsey et al., 2015). Polymers are the most used among the adhesive biomaterials for these purposes and they find application also in the design of scaffolds and dressing to restore tissue integrity and especially in tissue engineering to assure a deep and intimate contact between the systems and the lesions (Tarafder et al., 2020) (Dhandayuthapani et al., 2011) (Ikada, 2006a) (Li et al., 2024). In this regard, an outstand innovation for tissue engineering and regenerative medicine has been found in the fabrication of three-dimensional biocompatible (3D) scaffolds, which resemble the extracellular matrix and create a suitable microenvironment to restore tissues and organs (Mabrouk, 2020).

Bioadhesive polymers blended with different materials, such as metals, ceramics, and other functional polymers are used to develop tissue engineering scaffolds, as they improve the contact of the systems at the implantation site, enhancing tissue integration and reparation (Rana et al., 2023). In fact, bioadhesive polymers provide adhesion to biological components including cells, tissues, and organs by chemical or physical conjugation, avoiding scaffold dislocation once implanted in the body thus promoting the restore of tissue integrity (Wiltsey et al., 2015).

Given this premises, in this review, the mechanism of interaction between bioadhesive materials and biological surfaces will be described, underling the types of bonding (hydrogen bonds, hydrophobic interactions, electrostatic interaction, diffusion, physical interlocking and interpenetration) occurring at the interface when bioadhesive scaffolds and biological substrates (tissues or mucosae - mucoadhesion) are in contact. In addition, depending on the administration site, a variety of biological substrates that could represent different alternatives, including *ex-vivo* and artificial options, will be compared, and a discussion focused on the traditional and recently developed methods used to evaluate the bioadhesive properties of the different materials used in tissue engineering will be reviewed. To the best of our knowledge, the literature lacks a detailed and practical discussion of the procedures as well as a comparison of the *ex-vivo* and *in-vitro* biological substrates that could be employed by researchers to evaluate scaffolds bioadhesion.

Therefore, this review aims to fill this gap by providing an in-depth view on the most appropriate techniques (mechanical test, rheological synergism, zeta potential evaluation, ITC, AFM) to test scaffolds bioadhesive features based on the nature of the sample and the intended biomedical application. Subsequently, the most used biomaterials and in particular bioadhesives will be reported, with a focus on their use in tissue engineering and, more in details, in wound healing treatment.

## 2 Bioadhesion

The process of bioadhesion involves a series of molecular interactions occurring when two surfaces, with at least one is expected to be biological, come in contact over an extended period of time by spreading onto and wetting a biological surface and by forming physicochemical intermolecular interactions between the adhesive and the substrate (Khadem et al., 2022) (Khanlari and Dubé, 2013).

In biological systems, bioadhesion can be divided into three categories. Type 1 adhesion takes place between two biological substrates, as for example in the case of cell aggregation. Cell adhesion to culture dishes and biofilm formation on prosthetic devices and inserts are examples of type 2, which occurs when a biological component adheres to an artificial substrate. Lastly, type 3 adhesion describes the adhesion of an artificial material to a biological substrate, as in the case of the adhesion of polymers to soft tissues or skin (Mittal et al., 2020a) (Shaikh et al., 2011) (Lenaerts and Gurny, 2024) (Zate et al., 2010).

More in details, this phenomenon is ruled by different mechanism, such as chemical secondary bonds, as electrostatic bonds, and/or physical bonds, as interpenetration and interactions (Khanlari and Dubé, 2013).

### 2.1 Mechanism of bioadhesion

**Fig. 2:**
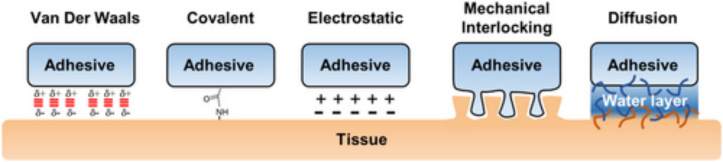
Schematics of the bioadhesive mechanisms. Reproduced with permission (Park et al., 2021).

Focusing on type 3 adhesion, a polymer takes advantages of interfacial forces to firmly bind with the biological substrate (Khadem et al., 2022). At this purpose, a combination of different interactions, including hydrogen bonds, hydrophobic interactions, electrostatic interactions, diffusion, physical interlocking and interpenetration, governs the bond between the bioadhesive and the adhesive substrate (Hamedi, 2022) (Khadem et al., 2022) (Fig. 2). More frequently, polymers firstly adhere to biological surfaces through interpenetration, followed by secondary non-covalent bonding, mostly hydrogen bonds (Mittal et al., 2020a). Interpenetration, as well as chain entanglement, are types of physical bonds based on the capacity of the adhesive and the adherent to interdiffuse into each other. The molecular structure flexibility and molecular weight of the bioadhesive control the entanglement of polymer chains (Wu and Zhao, 2023). In addition, mechanical interlocking occurs when bioadhesive compounds strongly and physically interact with the extracellular domains of transmembrane proteins, resulting in a strong interaction. In this case, it is important to consider the surface characteristics (such as the surface roughness) influencing the contact area and the interlocking (Park et al., 2021).

After the first interaction between the bioadhesive material and the biological substrate, chemical interactions, in particular non-covalent bonds, are built (Chen et al., 2021). Weaker and non-covalent bonds, such as hydrophobic interactions and hydrogen bonds, are most likely to be establish between the material and the adhesive surface in order to provide a fast adhesion (Mittal, 2020). In fact, chemical functional groups found in tissue-specific proteins, such as amines, carboxylic acids, hydroxyls, and thiols, interact with reactive groups in bioadhesives to generate intermolecular bonds (Wu and Zhao, 2023). Hydrogen bondings result from partial intermolecular interactions between a hydrogen atom bonded to a strongly electronegative atom, such as oxygen and nitrogen, and another electronegative atom with a single pair of electrons (Ding et al., 2024). For example, chitosan, a renowned bioadhesive material, is characterized by amine groups in its structure and therefore, hydrogen bonds are usually formed with various human body proteins and amino acids (Chen et al., 2021). Moreover, oppositely charged molecules on bioadhesive and tissue substrates generate electrostatic bonds, such as ionic bonds. For example, the negatively charged carboxylic acid groups on alginate chains form ionic bonding with divalent cation, such as Ca^2+^ (Ding et al., 2024). In this context, charge density is affected by the ionic concentration around the bioadhesive and determines the strength of the electrostatic attachment (Park et al., 2021). This underlines the influence of aqueous environment pH on these interactions as functional groups express different charges at specific pH values. For instance, amine groups are practically neutral and unable to interact electrostatically with a negatively charged polymer at pH values higher than 6.5 (Nakipoglu et al., 2023).

Alkyl chains and other non-polar hydrophobic compounds are involved in hydrophobic interactions. To promote interfacial adhesion, polymer chains having hydrophobic functional groups have a tendency to assemble and displace the interfacial water (Ding et al., 2024).

Alongside, covalent bondings generate strong connections and therefore, it is used to obtain a long-lasting and permanent adhesive feature. Reactive compounds such as isocyanates are commonly added into bioadhesives to produce covalent bonds with functional groups, that are found, as stated before, on the tissue surface, although this is not particularly recommended for the permanent modifications of the bioadhesive interface (Park et al., 2021).

In the development of tissue adhesives, a mix of covalent and noncovalent interactions is frequently employed, combining the characteristics of both interactions (Wu and Zhao, 2023). For example, this strategy was used to develop an adhesive hydrogel based on polyethylene glycol diglycidyl ether and a catechol-modified chitosan oligosaccharide. The high adhesion property of hydrogel is provided by a multitude of catechol, amino, and epoxy groups, further react with the tissues through both covalent and hydrogen bonds. More in details, covalent bonds are formed when the epoxy groups in the adhesive react with the amine and thiol groups of the nucleophilic amino acids in stratum corneum proteins (Robinson et al., 2008). Additionally, secondary interactions are established with tissues through the groups of quinones and catechol hydroxydes (Jing et al., 2017).

### 2.2 Mucoadhesion

Mucoadhesion is a type 3 adhesion and is based on the interaction between an artificial substrate and a biological substrate covered by a mucus layer namely a mucous membrane (mucosa) (Woodley, 2001) (Shaikh et al., 2011).

The major component of mucus is mucin, a highly glycosylated glycoproteins, capable to retain water molecules and to form viscoelastic gel, firmly layered onto the mucosa surface (Asati et al., 2025) (Weston et al., 2023) (Prasher et al., 2022). Mucin structure is characterized by a protein backbone and side chains, formed by oligosaccharides, such as galactose, fucose, *N*-acetylglucosamine, *N*-acetylgalactosamine, and sialic acid. The structure of mucin and in particular the types of oligosaccharides residues are influenced by the specific physiological district. Moreover both sulfated sugars and sialic acid are completely ionized at pH higher than 3, which gives mucin a net negative charge and the propensity to electrostatically interact with cationic biomolecules (Thirawong et al., 2008). Thus, the degree of adhesion and the residence time of a mucoadhesive material is generally influenced by pH, ionic strength, mucin type (composition of the oligosaccharide chains), which have a significant impact on the interactions between mucins and polymers (Russo et al., 2016).

**Fig. 3:**
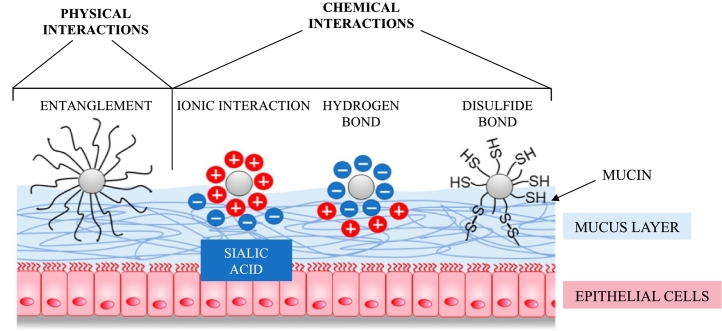
Graphical representations of the mucoadhesive mechanisms, underlining chemical and physical interactions with mucus layer. Adapted from (Sato et al., 2023) CC BY 4.0.

Different stages and types of bonding interactions are identified to describe the process of mucoadhesion (Gawas et al., 2016) (Patel et al., 2000) (Fig. 3). Firstly, a close physical contact between the material and the mucosa is established during the contact stage. This is followed by the interpenetration step, due to the interdiffusion of the chains of the mucoadhesive polymer and the mucin glycoproteins, resulting in chains entanglement (Brahmbhatt, 2017) (Hamedi, 2022). The strength of the mucoadhesive joint is strictly related to the thickness of the mucoadhesive interface due to the reciprocal diffusion of the mucoadhesive polymer and the mucin chains (Mittal et al., 2020a). When the consolidation phase occurs, mucus and mucoadhesive polymers interact because of secondary bonding formation, specifically hydrogen bonding and van der Waals attraction. The density of these bonds depends on the chemical structure of the polymers and is directly related to strength of mucoadhesive joints. In fact, although the strength of the mucoadhesive joint due to weak reversible chemical bond is negligible, multiple bonds are able to confer high adhesion force. Therefore, the chemical groups of mucoadhesive polymers at the interface promote mucoadhesion and are mainly hydroxyl, carboxyl, amino, and amide groups. Strong hydrogen bonding groups and cationic charges, a high molecular weight, suitable chain flexibility, surface energy properties and facilitated spreading onto mucus are all crucial polymer characteristics crucial for mucoadhesion (Thirawong et al., 2007). Simultaneously, mechanical interactions, such as entanglement between polymer and mucin chains reinforce the mucoadhesive bonds and prolong adhesion (Ahmady and Abu Samah, 2021).

As mentioned, positively charged polymers, such as chitosan, are characterized by mucoadhesive behavior based on multiple mechanisms, including hydrophobic contact, hydrogen bonding, and electrostatic interaction with the negatively charged oligosaccharide residues on mucin moieties (Alcantara et al., 2022).

## 3 Biological substrates employed during the investigation

To mimic the physiological scenario, test conditions should be as similar as possible to the application site of the bioadhesive system, as any variations could tune the bioadhesion (Amorós-Galicia et al., 2022).

In particular temperature, environmental pH and surface properties should be considered to target a variety of sites, as for example cutaneous, buccal, nasal, ophthalmic, rectal, vaginal and cardiac (Ikada, 2006b). For instance, skin epidermis includes a lipidic barrier and it is an air liquid interface; on the contrary, the mucosal epithelium of the intestine is exposed to an aqueous environment and to variable pH (Amorós-Galicia et al., 2022).

For this reason and because of the lack of a standardized method of evaluation, various biological substrates have been developed to assess bioadhesion and mucoadhesion, mainly through *in-vitro* and *ex-vivo* tests (Mittal et al., 2020b) (Table 1).

**Table 1:** Tissues and artificial substitutes employed during the evaluation of bioadhesion and mucoadhesion.

Target site	*Ex-vivo* tissue	*In-vitro* alternative	REF
Skin	Porcine skin (Pig ear skin)	Skin-like substitute	(Dick and Scott, 1992) (Pan et al., 2023) (Albarkah et al., 2015)
Vaginal mucosa	Cows vaginal mucosa	Mucin dispersion + simulated vaginal fluid	(Lorenzo-Veiga et al., 2020) (Eldesouky et al., 2021) (Marxen et al., 2017)
Intestinal mucosa	Pig intestinal tissue Rat model Sheep small intestine	Human intestinal epithelium using Caco-2 cells	(Drori et al., 2025) (Fürst et al., 2019) (Drori et al., 2024)
Buccal mucosa	Pig oral mucosa	Mucin dispersion + simulated saliva Human buccal epithelium based on the standard buccal cell line (TR146)	(Fürst et al., 2019) (Drori et al., 2024)
Ophtalmic mucosa	Bovine cornea	Mucin dispersion + simulated lachrymal fluid	(Bhushan and Tang, 2011) (Lir et al., 2007)

### 3.1 *Ex-vivo* tissues

Tissues from mice, pigs, and sheep have been proposed in literature to assess *ex-vivo* bioadhesion (Drori et al., 2025) (Hickman et al., 2017) (Kocsis et al., 2022). Porcine skin is the primary model for human skin, as it exhibits structural, histological and physiological similarities (Amorós-Galicia et al., 2022) (Kocsis et al., 2022; Jacobi et al., 2007). In particular, pig ear skin is considered the most suitable alternative to human skin, as demonstrated by the qualitative and quantitative tests to correlate the tissue physiological and histological structure, including hair follicles and skin layers, with human skin (Jacobi et al., 2007) (Dick and Scott, 1992).

In case of mucoadhesion, a mucosal epithelium, excised from animal mucosa, is employed. At this purpose, the literature suggests cows, pigs or rat mucosae (Amorós-Galicia et al., 2022) considering epithelia derived from different districts, such as vaginal, intestinal or nasal mucosa depending on the administration (Mittal et al., 2020b). In particular, pig intestinal tissues and oral mucosa, including pig oesophageal mucosa, have been used in some research because they resemble human intestines or buccal tissue in both morphology and physiology (Diaz del Consuelo et al., 2007) (Pan et al., 2023) (Fürst et al., 2019). However, other authors have selected mouse models due to their mucosal and genetic similarities to humans (Drori et al., 2024), even though, there are evident differences as the case of buccal mucosa that is keratinized, unlike the human one or skin with high density of hair follicles (Patel et al., 2012). Sheep is also a selected species although its digestive system greatly differs from human (Flores Tracey et al., 2018). Cows vaginal mucosa has also been extensively used as a model substrate due to the high degree of similarity with human vaginal mucosa (das Neves et al., 2008) (Baloğlu et al., 2003). Bovine and pig cornea and conjunctival mucosa are commonly used to evaluate mucoadhesive properties of ophthalmic formulations (Lorenzo-Veiga et al., 2020) (Eldesouky et al., 2021).

### 3.2 Artificial alternatives

Due to their less ethical concerns, lower costs, availability and ease of use, *in-vitro* models, that do not require the use of excised animal tissues, are typically chosen (Amorós-Galicia et al., 2022). There are several significant benefits of using an appropriate artificial model substrates in place of actual excised tissues. Moreover artificial substrates allow more reliable data due to less variability and inconsistency that in *ex-vivo* substrates is quite common due to the individual variations of the tissues (Bhushan and Tang, 2011). In the cases of skin adhesion, a skin-like substrate mimicking the surface and mechanical characteristics of human skin is optimized. The model substrate has been validated and guarantees results in correlation with human skin (Lir et al., 2007) (Bait et al., 2011). Specifically, this skin substitute is based on gelatin and fatty components, and replicates mechanical and interfacial characteristics of normal skin (Bait et al., 2011). Similarly, mucin dispersion is employed to assess mucoadhesion, as model mucus (Thirawong et al., 2008) (Lee et al., 2016). Porcine gastric mucin is generally preferred and mucin dispersion in suitable concentration and buffer allows to simulate different biological fluids typical of specific districts as it is the case of simulated vaginal fluid, simulated lachrymal fluid or simulated saliva (Albarkah et al., 2015) (Falavigna et al., 2020) (Guarise et al., 2023) (Marxen et al., 2017). More interestingly, an *in-vitro* model for the characterization of bioadhesion has been developed using Caco-2 cells, able to create a monolayer of polarized cells with tight junctions and microvilli, with a morphology comparable to that of human small intestine (Drori et al., 2025). Furthermore, to mimic human buccal epithelium, an *in vitro* model based on the standard human buccal epithelial cells, TR146, was developed as buccal *in-vitro* substrate, as a valid alternative to porcine buccal tissue and oesophageal tissue, which are employed for *ex-vivo* permeability and absorption studies. To better simulate the buccal environment, after the growth of the cells on an elastomer support, a mucous layer, composed of mucin, was added (Rahamim et al., 2024).

## 4 Characterization of bioadhesion

Several techniques are used to evaluate, both quantitatively and qualitatively, the adhesive properties of scaffolds. The techniques used are normally based on different principles and in some cases are associated giving complementary information.

### 4.1 Mechanical tests

#### 4.1.1 Tensile test

**Fig. 4:**
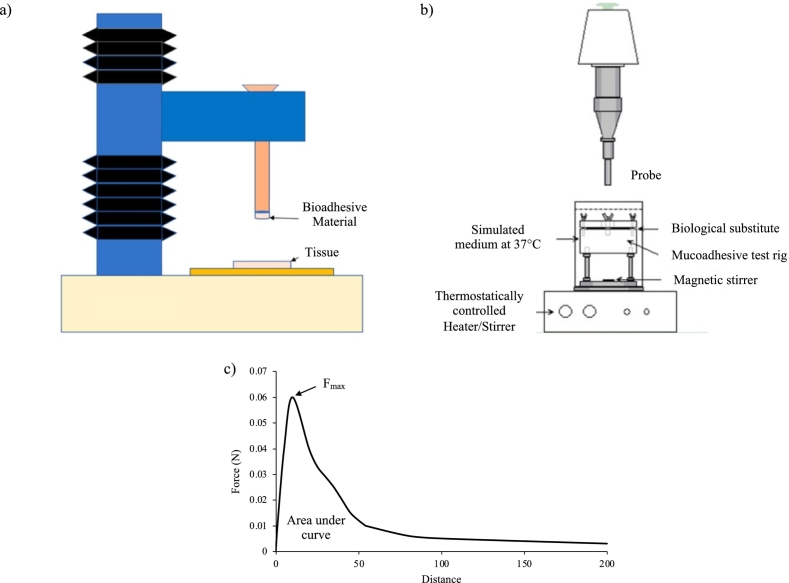
a) Schematic representation of a dynamometer used for the evaluation of bioadhesive materials. Adapted from (Amorós-Galicia et al., 2022) CC BY 4.0. b) Schematic representation of a dynamometer equipped with a mucoadhesive rig. Adapted with permission from (Woertz et al., 2013). c) Typical output graph obtained from a tensile test, showing force *versus* distance data. The area under the force *versus* distance curve represents the work of adhesion, and the height of the peak represents the greatest force needed to remove the probe from the tissue (maximum detachment force; Fmax).

Tensile test is a mechanical test frequently used to measure the force required to displace the bioadhesive interface based on the adhesive material and the biological substrate (Thirawong et al., 2007) (Bartlett et al., 2023). A dynamometer equipped with a test rig, is employed to measure the force needed to detach the interface (Fig. 4-a) (Pagano et al., 2022). The device is placed under the probe onto the basement platform of the equipment (Fig. 4-b). The test rig is composed by a lower disc where the biological substrate, such as tissue or mucosa or simulated biological substrate, is hold. On top of it, another disc of the device, which present a hole in the center, is located and fixed with screws onto the other disc (Woertz et al., 2013). The test sample is applied onto the probe connected to the movable arm of the dynamometer. The test sample and the biological substrate are put in contact normally under a predetermined preload for a scheduled time and the force needed to detach the interface is recorded upon the climb back of the probe until the two faces are split and the adhesive joint breaks. During the test, the sample and the biological substrate can be heated to a predetermined temperature using a suitable heating bath and the temperature could be set to simulate a specific physiological temperature (skin and eye 32 °C; buccal, nasal and vaginal: 37 °C) (Woertz et al., 2013). The output of the measurements is typically a plot where force of detachment is function of the displacement of the interface as reported in Fig. 4-c. As critical parameters, it is possible to identify the maximum force needed to separate the bioadhesive device from the substrate surface that correspond to the peak force Moreover it is possible to obtain the work of adhesion, calculated as the area under the force *vs* distance curve (Thirawong et al., 2007). It is also possible to obtain normalized parameters considering the contribution given by the sample consistency onto the bioadhesive phenomenon. This is estimated by measuring the bioadhesion without the biological substrate and calculating the ratio between the difference between the values obtained for the sample in presence and without the biological substrate and the value obtained without the biological substrate as follows:

$$\mathrm{\Delta F}=\left(F_{\max\;\mathrm{bio}}-F_{\max\;w/o-\mathrm{bio}}\right)/F_{\max\;w/o-\mathrm{bio}}$$

$$\mathrm{\Delta W}=\left(W_{\mathrm{bio}}-W_{w/o-\mathrm{bio}}\right)/W_{w/o-\mathrm{bio}}$$

where Fmax is the maximum force of adhesion; W is the work of adhesion; bio is parameter obtained with biological substrate; w/o-bio is parameter obtained without biological substrate.

#### 4.1.2 Peel adhesion test

**Fig. 5:**
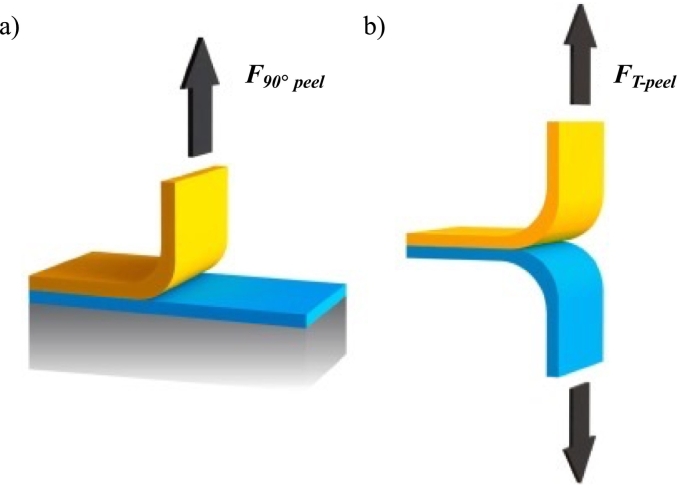
Schematic representation of the peel adhesion test set-up: a bioadhesive material is attached to the fixed surface and it is peeled in a) 90° or b) T-peel set up. Adapted with permission from (Bartlett et al., 2023).

Alongside with tensile test, another commonly used mechanical method is peel adhesion test. This test is particularly used to characterize the ability of adhesives to withstand peeling off a biological surface (such as skin) over time. 5Fig. 5 reports the schematic representation of the experimental set up.

Different approaches based on peeling model have been developed to meet various surface contact scenarios. The most popular technique is based on the measurement of the force needed to remove an adhesive sample from the biological substrate using a specific removal angle. In particular, among those, 90° peel test is frequently used. T-peel test (also named 180° peel test) is an evolution of the peel test and is performed detaching two flexible substrates (same dimensions) adhered together: in particular one substrate protrudes vertically with respect to the other and the free end parts of both are firmly blocked using grips (Gu et al., 2016).

As for tensile test, the force of detachment and total work of adhesion, applied to break the adhesive bond, can be measured to evaluate the interaction (Liu, 2018).

#### 4.1.3 Lap shear test

**Fig. 6:**
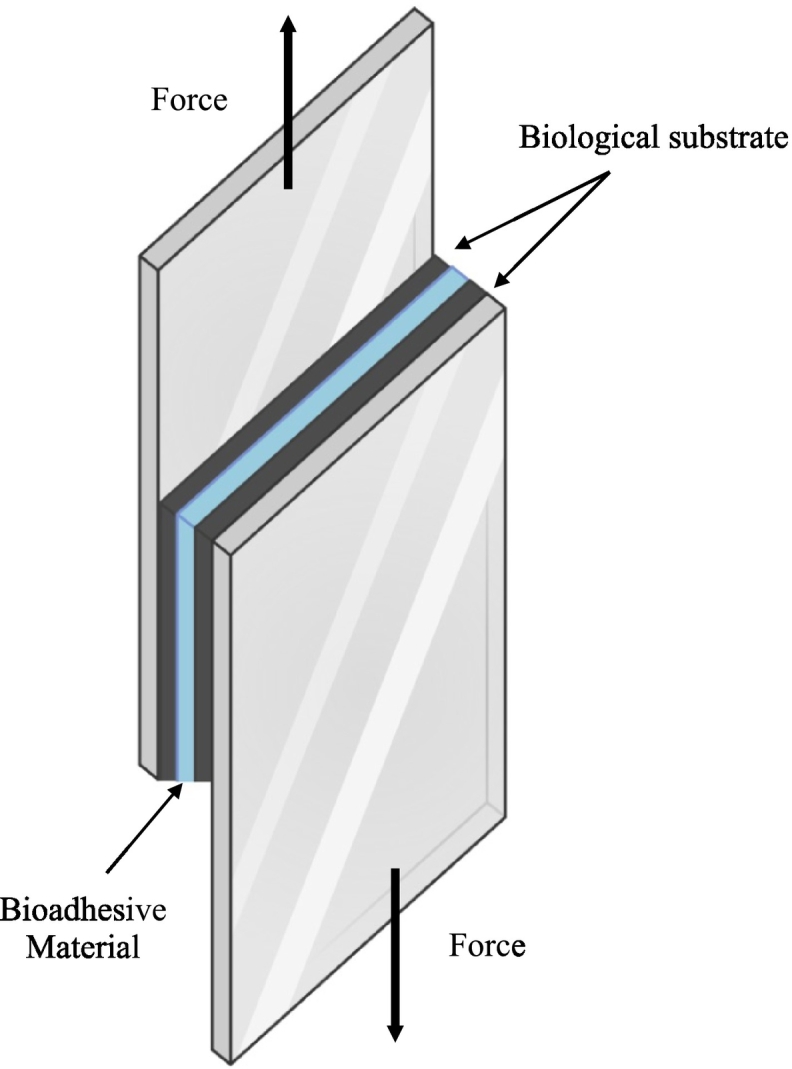
Schematic representation of the lap shear test set-up. Adapted with permission from (Si et al., 2023).

As reported in Fig. 6, lap shear refers to a method of testing the adhesive strength of bonded materials by applying a tensile force parallel to the bond line. Two biological substrates are bonded together with a bioadhesive, with a portion of the materials overlapping (the “lap”). Then a tensile force is applied to the bonded materials, pulling them apart along the bond line. The force required to cause failure (separation of the interfaces) is recorded, and the lap shear strength is measured as the failure stress in the adhesive, which is calculated by dividing the failing load by the bond area (McDermott et al., 2004). The lap shear strength is actually a shear stress at break, also known as adhesion strength at break, and quantifies the adhesiveness (Serrero et al., 2011). A preload could be applied on the adhesive joint for a predetermined time, to favor the formation of the adhesive bonds, and removed after a predefined time (McDermott et al., 2004).

Analogous evaluation could be performed using a dynamic mechanical thermal analyzer (DMA), normalizing the force to overlap area (Ghovvati et al., 2022) (Chen et al., 2006). DMA is a highly-accurate, precise, and non-destructive characterization method, that could apply different loading modes, such as tension, torsion and compression to better characterize the mechanical behavior of the adhesives (Jaramillo-Isaza et al., 2022).

#### 4.1.4 Applications

Tensile test is profitably used to assess bioadhesion towards *ex-vivo* biological substrates. A texture analyzer is equipped with the A/MUC rig where the biological sample is blocked while the samples are applied onto the probe of the movable arm.

The influence of the molecular weight on chitosan gel bioadhesion has been assessed using the inner part of pig ear; hydrogel based on carbopol, a synthetic bioadhesive, was used as a reference, in this study. Chitosan was able to persist 2 folds longer on the skin than Carbopol based hydrogel (Hurler and Skalko-Basnet, 2012). Bioadhesion of solid systems was also assessed using the same set up. In particular, chloramphenicol loaded PVP nanofibers have been evaluated and the tests showed that the presence of PVP increased the nanofibers bioadhesion, potentially influencing the drug release from the scaffold (Tamm et al., 2016).

The *in vitro* adhesion of solid films, based on hydroxypropyl cellulose, manufactured utilizing semi-solid extrusion 3D printing have been also assessed (Sjöholm et al., 2020). Force and work of adhesion were tested using a Texture Analyzer and *in vitro* skin substrate (Sjöholm et al., 2020).

As for mucoadhesion, porcine gastric mucin proves to be successful in the assessment of the formulation mucoadhesion. For example, its use is reported in the development and characterization of a mucoadhesive *in-situ* gelling formulation based on κ-carrageenan and hydroxypropyl cellulose for the management of esophagitis and oral mucositis. An artificial saliva was prepared and used as a solvent for mucin and the dispersion was then employed as substrate for tensile test (Vigani et al., 2019a). Similarly, a dispersion of mucin in a vaginal simulating fluid was prepared and employed to assess the mucoadhesive features of a gel to prevent candidiasis by prolonging the permanence of *Lactobacilli*, as therapeutic agents (Vigani et al., 2019b).

Peel adhesion test has been used to evaluate the adhesive strength of various semisolids based on gelatin (Gowda et al., 2020).

Lap shear has been used to fully characterize tissue adhesive properties of a poly (vinyl alcohol)-borax hydrogel enriched with tannic acid-coated silk microfibers. Interestingly, different biological substrates were employed and in particular porcine skin, finger epidermis and different mouse organs. After, the hydrogel was applied to the skin, tests for bending, stretching, and torsion were conducted to further examine the robustness of the method. Moreover, the same characterizations were carried out on samples after 24 h at room temperature in phosphate buffer saline (PBS) to assess if the hydrogel was able to stably adhere to the biological substrate at wet state. Extensive stretching, bending, and twisting tests have demonstrated that the hydrogels stick firmly to the pig skin both before and after a 24-h PBS treatment. This can probably be explained by the presence of catechol groups of tannic acid, which binds thiol or amino groups, present on the tissue, and enhance hydrogel bioadhesion (Si et al., 2023).

### 4.2 Rheological synergism

**Fig. 7:**
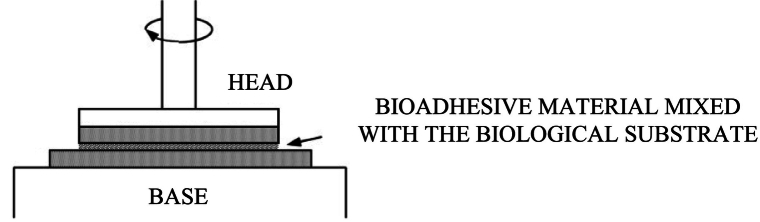
Schematic representation of the lap shear test set-up. Adapted with permission from (Wang and Kornfield, 2012).

Rheological synergism is a technique that allows to characterize the viscoelastic properties of the bioadhesive interface and in particular the modifications in the viscosity of both adhesive material and the biological substrate that are likely to result from conformational alterations, physical entanglement, and hydrogen and van der Waals bonds (Silva et al., 2017). Fig. 7 reports the set up used for the measurement of rheological synergism using rotational rheometer equipped with plate-plate combination.

This technique has been developed for the evaluation mucoadhesion, however it is also used to measure the interaction with physiological proteins such as collagen, the main component of the extracellular matrix (ECM). Several investigations proved that the bioadhesive interface possesses peculiar properties such as the resistance to deformation (Madsen et al., 1998). The viscosity (η) and viscoelasticity (G' elastic and G" viscous muduli) of bioadhesive solution/dispersion, biological substrate (mucin or ECM proteins) and the mixture of the two components having the same original concentrations are measured and the rheological synergism is calculated as the difference between the viscosity or G' values of the adhesive and the biological substrate mixture at the same shear rate or frequency, respectively, and the sum of the viscosity or G' values of each components at the same concentration of the mixture (Sriamornsak and Wattanakorn, 2008).

It is possible to evaluate rheological synergism using the bioadhesive substrate and mucin at different weight ratios (Silva et al., 2017).

#### 4.2.1 Applications

Rheological synergism is considered a quantitative analysis of the bioadhesive properties of materials as a higher ΔG′, or Δη indicates stronger interactions (de Oliveira Cardoso et al., 2020). The investigation is possible only with semisolid materials and this is the reason why no *ex-vivo* substrate is considered while is very common the use of the mucins in dispersion. Formulations giving a positive synergism are thought to have more capacity to interact with the substrate (Vigani et al., 2019a), while negative synergism suggests a strong interaction that causes a complex formation and therefore precipitation (Rossi et al., 2014) (Valentino et al., 2023). Specifically, in literature, this method is particularly used to assess mucoadhesion. Different polymer/mucin ratios can be used as it was proved that different mucoadhesive behaviours result from the amount of mucin. For example, it was found that viscosity increases synergistically at higher chitosan/mucin weight ratios, due to the physical chain entanglements between polymer and mucin chains which are responsible for the mucoadhesive bond (Rossi et al., 2001). However, the chitosan-mucin mixtures prepared using weight ratios lower than 1:2 result in negative rheological synergism values because of the precipitation of a mucin-chitosan complex which induced the mixture viscosity to decrease (Rossi et al., 2014) (Rossi et al., 2001).

### 4.3 Zeta potential

Zeta potential is a specific property of a surface, and it is related to the polarization, or the charge densities present onto the bioadhesive and their modifications following the interaction with the biological substrates. The surface properties are important for understanding and controlling the interface processes. Generally these are tuned also by the presence of electrolytes in the environment since ionization and consequently by the surface charge is strictly affected by the environmental pH (Ferraris et al., 2018) (Berthold et al., 1996).

In case of nanoparticles, zeta potential is assessed using electrophoretic light scattering (ELS) able to evaluate electrophoretic mobility of the charged particles dispersed in a liquid under an electric field (Varenne et al., 2019). In particular, particles having a positive zeta potential move in the direction of the negatively charged electrode. On the contrary, negatively charge particles are attracted by the positive electrode (Xu and Scarlett, 2002) (Strand et al., 2001). If a bioadhesive joint occurs, the zeta potential of a colloidal dispersion is likely to reverse upon mixture with the bioadhesive (Andreani et al., 2015).

In case of wide surface, zeta potential is assessed using the streaming potential method. This is based on the formation of an electrical double layer onto the surface due to the presence of an electrolyte solution, and the difference of potential between the two layers is measured (Duy Thanh, 2018) (Metwally and Stachewicz, 2019; Dillmann et al., 2020). The measurements of zeta potential as a function of pH enable to evaluate the systems isoelectric point (IEP) that allow to predict the type of surface charges (positive or negative) in a specific medium (Polak et al., 2023).

#### 4.3.1 Applications

Zeta potential is of paramount interest to predict the cells capability to adhere to a specific 3D construct for homing. In fact, as cells membrane is negatively charged, positive zeta potential of scaffolds for tissue engineering promotes the cells spreading and growth to rebuild the damage tissue (Metwally and Stachewicz, 2019) (Chen et al., 2011), as for example, polyurethane membranes modified by negatively charged polyelectrolytes as polyacrylic acid, heparin, alginate, dextran sulfate, chondroitin sulfate. A sodium salt was not able to allow, *in-vitro*, the adhesion of human endothelial cells conceivably due to the electrostatic repulsion. However, when collagen was used as membrane coating and the surface charges reverse cells adhesion occurred (Zhu and Sun, 2004), as well as proteins adsorbed onto the positively charged surfaces, consequently causing the formation of integrin connections, and focal adhesion points (Metwally and Stachewicz, 2019) (Ruggeri et al., 2023).

In another work, chitosan films have been tested as mucoadhesive and the properties were related to mucin dispersion pH change due to mucin-chitosan interaction (García et al., 2017).

Analogously, nanoparticles behavior and in particular, as example, chitosan and hyaluronic acid nanoparticles proved to form mucoadhesive joint between the positively-charged amino groups of chitosan and the negatively-charged sialic acid residues of mucin. Due to the presence of chitosan, nanoparticles zeta potential values indicated a notably positive surface charge. After the incubation with mucin, a significant decrease in zeta potential was registered because of the ionic interactions of negatively charged mucin particles onto the surface of positively charged chitosan nanoparticles, as shown in Table 2 (Silva et al., 2017).

**Table 2:** Values of zeta potential of mucin dispersion 2 mg/mL, chitosan and hyaluronic acid nanoparticles (NP), NP with the addition of (hydroxypropyl) methyl cellulose (HPMC) (NP eye drop formulation), and HPMC hydrogel 0.75 % (*w*/*v*) upon dilution 1:1 with mucin dispersion 2 mg/mL (Silva et al., 2017).

Solutions	Mucin 2 mg/mL
Mucin 2 mg/mL	−31 ± 1
Empty NP	+ 12 ± 0
Empty NP eye drop formulation (HPMC 0.75 %)	+ 7 ± 0
HPMC 0.75 %	−9 ± 1

### 4.4 Atomic Force Microscopy (AFM)

**Fig. 8:**
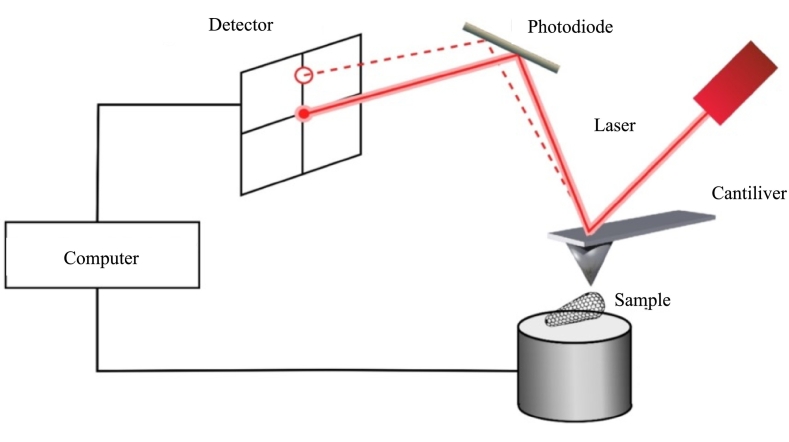
Schematic representation of AFM set-up. Adapted from (Rousso and Deshpande, 2022) CC-BY 4.0.

AFM is a powerful technique capable of assessing the surface topography. A cantilever scans the sample and its deflection due to the peak and valley results in the 3D reconstruction of the surface. It is also possible to record the attractive and repulsive force developed between the cantilever tip and the sample surface (Pierce et al., 1994) (Butt et al., 2005) (Eaton and West, 2010) (Patel et al., 2000). A laser diode beam is focused on the free end of the cantilever, and the reflected beam position is recorded by a detector providing high-resolution 3D images (Dufrêne, 2002) (Kar, 2012), as reported in Fig. 8.

When the cantilever tip is modified using a suitable biological substrate (such as a protein) the interaction with a biopolymer filmed over a specific surface is assessed by means of the increase of the attractive forces during a force-distance (Pierce et al., 1994). (Gunning et al., 2013). The bioadhesive joint is subjected to a gradually decrease of the attractive force due the subsequent tip-surface retraction until the bioadhesive interface breaks at a critical force (Hinterdorfer et al., 2002).

#### 4.4.1 Applications

AFM is able to detect the change in system topography due to the formation of the bioadhesive interface. In particular the presence of aggregates due to complexation is visible as in the case of the interaction between mucin and chitosan where long linear filamentous characteristic of both chitosan and mucin assumed a entangled structure giving rise to a globular structure (Deacon et al., 2000).

Analogously, this was assessed also in the case of the interaction between mucin and pectin with chain association and the consequent hydrogen bonds formation (Sriamornsak et al., 2010).

Similarly, AFM was employed to evaluate the presence of adsorbed bioadhesive on mucosal cell surfaces. In a study, buccal cells were treated with a solution of hydroxypropyl methylcellulose, chitosan, or polycarbophil. While the surfaces of untreated cells appeared smooth, with tiny holes and indentations, the interaction with the polymers increased the surface roughness, suggesting the polymer chains adsorption on the cell surfaces (Patel et al., 2000). AFM also allows to measure the adhesion forces if a bioadhesive is attach to the cantilever tip (Pierce et al., 1994). In fact the binding causes a rising force subsequent to tip-surface retraction due to the bond breaks at a critical force (Hinterdorfer et al., 2002). This approach was employed to compare the bioadhesive behavior of alginate and chitosan, covalently attached on the tip, when in contact with mucin (Haugstad et al., 2015). Similarly, AFM was employed to quantitively investigate the interaction between polydopamine-grafted alkali lignin nanocapsules suspension and pigskin, which served as the organic substrate surface (Zhou et al., 2020).

### 4.5 Isothermal Titration Calorimetry (ITC)

ITC is a highly sensitive technique able to assess heat transfer caused by formation and dissociation of a molecular complex when the bond between the interacting molecules from the free to the bound state and *vice versa* determines heat absorption or emission (Ghai et al., 2012).

During the analysis, one binding partner in the sample cell is titrated, with several addition, with the other binding partner. The heat transfer is measured during each injection and is defined as the electrical power needed to maintain the minor temperature differential between the sample and reference cells (Jelesarov and Bosshard, 1999). As the binding is saturated the heat signal gets closer to zero (Pilipenko et al., 2021). The temperature and buffer used suit the specific binding system under investigation, allowing also to mimic physiological conditions (Duff et al., 2011). The binding curves allow to calculate the stoichiometry of the binding (Xu et al., 2015).

There are many evidences in literature focus on bioadhesion, and more specifically on mucoadhesion (Xu et al., 2015) (Liu et al., 2022a) (Zandanel et al., 2021).

#### 4.5.1 Applications

ITC is a powerful technique to evaluate the binding constant and to quantify the bioadhesion, however the experiment conditions greatly affect the results since the binding is sensitive to variations in temperature, solvent composition, ionic strength, pH, and other environmental factors (Liang, 2008) (Velázquez-Campoy et al., 2004). In this context, the mucoadhesive properties of chitosan has been studied at different pH values. In fact the formation of electrostatic interactions usually occurs in the pH range between the polymer pKa value and the protein isoelectric point, with an ideal pH being reached when all the molecules have the same number of charges.

In a study, porcine gastric mucin was representative of the oral mucosa and two pH were used to perform the experiment: pH 6.3 to mimic the physiological pH of the mucosa and pH 5.2 selected as it is theoretically associated with a higher affinity between chitosan and mucin (Menchicchi et al., 2014) (Meng-Lund et al., 2014). Electrostatic interactions between the positively charged glucosamine residues of chitosan and the negatively charged sialic acid residues on the mucin at lower pH, have been proved and in particular, a higher association constant was obtained at pH 5.2 compared to pH 6.3, indicating that the interaction is pH dependent, and it is enhanced at lower pH (Meng-Lund et al., 2014). The thermodynamic parameters of biomolecular interactions, such as affinity, enthalpy, entropy, and stoichiometry, are furthermore fundamental to better understand the nature of the interaction (Dutta et al., 2015). In the previously mentioned study, it was possible to claim that the reaction between chitosan and porcine gastric mucin was entropy driven as enthalpy was positive and therefore unfavorable (Meng-Lund et al., 2014).

The same approach was employed to investigate the mucoadhesion of eparin-*graft*-poly(N-isopropylacrylamide) and chondroitin sulfate-*graft*-poly(N-isopropylacrylamide) copolymers. The results prove that an exothermic reaction occurs between the species, which was probably due to the formation of hydrogen bonds (Pilipenko et al., 2021).

## 5 Critical comparison of the techniques and biological substrates

### 5.1 Nature of the samples

Based on the nature of the bioadhesive and the application/administration district, the most appropriate tests among the ones described should be selected (Table 3). Among the systems for the treatment of cutaneous lesions different formulations can be considered as semisolids, such as hydrogels, solid platforms, such as films, nanofibers or sponge-like dressings, or liquid, as nanoparticles dispersion. Mechanical tests are mostly employed to evaluate bioadhesion of semisolids or solids. On the contrary, in case of nanoparticles, it is possible to perform ITC and ELS, hile rheological synergism can be used to characterize both semisolids and liquid systems. Interestingly, AFM can be adapted to perform analysis on solids, semisolids and liquids.

**Table 3:** Classification of bioadhesive tests on the basis of the type of sample.

Solid	Semisolid	Liquid
Mechanical test (tensile test, peel adhesion, lap shear)	Mechanical test (tensile test, peel adhesion, lap shear)	Zeta potential (Electrophoretic Light Scattering)
Zeta potential (electrokinetic methods-Surpass3)	Rheological synergism	Rheological synergism
AFM	AFM	AFM
		ITC

### 5.2 Techniques

The most versatile and used technique is the tensile test, which proved to be adaptable to different formulations, such as solids (minitablets and pellets) and semi-solid (emulsions) (Silva et al., 2017). However, minor changes in the set-up parameters can result in deviations, therefore requiring the same conditions for all measurements. Specifically, the test can be influenced by pre-test contact time and applied force between material and substrate. In particular, as reported in literature, the bioadhesive strengths of chitosan, alginate and gelatin hydrogels in contact with porcine buccal tissue or oesophageal tissue were measured using two different pre-test contact times (120 s and 720 s) and applied forces (0.02 N and 0.05 N). The bioadhesive tensile strength resulted directly dependent to both those variables (Rahamim et al., 2024). In an another study different pre-test contact forces (0.3, 0.5 and 1 N) were used and the higher tensile strength was found in the case of 0.5 N, while a further increase up to 1 N in pre-test load caused a decrease of the tensile strength. Similarly, lower (15 s and 20 s) and higher (900 s) pre-test contact times were discarded as they either resulted in negative values or large deviations; on the contrary, 60 s was selected as the most suitable time to achieve reliable and consistent results. In addition, the probe withdrawal speed should also be considered as it affects data variability. Therefore, when developing a standardized methodology, contact time, pre-test force and the withdrawal speed should be carefully evaluated and higher than 60 s, 0.5 N, and 0.1 mm/s, respectively (Amorós-Galicia et al., 2022). To reduce the influence of those variables, work of adhesion (area under a force-distance curve) could be calculated and compared, as this is the less sensitive parameter to measurement variables and only contact time was found to affect the results (Bassi da Silva et al., 2018).

Another method is the rheological synergism, which, however, is highly influenced by the bioadhesives characteristics including physicochemical and structural ones, particularly the viscosity, limiting its reliability. While tensile test is able to characterize stronger adhesion resulted by the increase of gel strength, positive rheological synergism values were obtained with weak gels, characterized by low viscosity and G' values (Hägerström and Edsman, 2003). Moreover, when performing the analysis, standard conditions such as bioadhesive concentrations and ratio and type of biological substrate, should be considered. Specifically, by increasing mucin concentration, an enhancement of the bioadhesive properties occurred, due to the higher availability of mucoadhesive binding sites and higher interactions with the bioadhesive polymer, thereby maximizing the mucoadhesive properties. However, additional increases in mucin concentration conceivably lead to the saturation or even a decrease in the observed effect. These findings suggest the need of investigating the stoichiometry of the interaction between a bioadhesive material and the biological substrate, in order to achieve their bonding (Sriamornsak and Wattanakorn, 2008).

A correlation was also found between tensile test and zeta potential. In a study, a higher tensile force was corresponding to a higher zeta potential, proving that these measurements could be used to confirm the materials bioadhesiveness when the electrostatic bonds rule the bioadhesive interaction. However, zeta potential measurements limits the use of higher sample concentrations, influencing the mucoadhesive measurements. Furthermore for some samples, zeta potential could be also influenced by environmental factors (buffer capacity and ionic strength), often limiting the technique reliability (Bogataj et al., 2003).

### 5.3 Biological substrates

As previously mentioned, *ex vivo* animal tissue is the biological substrate of primary choice, and occasionally the only method used to evaluate and validate bioadhesion and mucoadhesion.

In case of buccal bioadhesion, porcine buccal tissue and oesophageal tissue are the most employed substrates as they mimic the histological properties and composition of the human epithelium. Those substrates were studied in terms of bioadhesive strengths when in contact with gelatine, chitosan and alginate hydrogels. The findings suggested that porcine oesophageal tissue expressed a higher bioadhesion interaction (1.8 to 2.6 times higher) compared to porcine buccal tissue and this could be probably due to the rugosity of the oesophageal tissue given by plicae. To reduce the use of *ex-vivo* substrates, *in-vitro* models have been developed; however, studies are mandatory to validate the consistency and reliability of these alternatives. At this purpose, an *in-vitro* alternative, based on a buccal epithelium cell layer with and without the addition of a mucin dispersion on the top, was developed. The cell layer without the addition of mucin could be a valid alternative to predict buccal adhesion as it revealed similar bioadhesive values to the ones obtained with porcine oesophageal tissue (Rahamim et al., 2024).

An *in-vitro* alternative to evaluate mucoadhesion is the use of mucin solutions to limit the use of mucosa and specifically porcine mucosa as they present comparable histological features to humans. However, when comparing the two substrates using the same test parameters, differences were recorded in the results, requiring the fine tuning of the parameters to optimize the analysis (Woertz et al., 2013).

## 6 Biomaterials in tissue engineering and wound healing

In regenerative medicine, bioadhesion is crucial to create a continuity between the scaffolds and the surrounding tissues where the cell recruitment comes from (Capella et al., 2019) and biomaterials are fundamental components to sustain and promote cell homing and proliferation and ECM production. In fact, bioadhesives favor the direct contact between the formulation and the application/administration site to increase the active ingredient bioavailability in case of local release or to maintain an intimate contact with the surrounding tissue and favor tissue integration (Khanlari and Dubé, 2013) (Xiong et al., 2021) (Yu et al., 2022) (Saha et al., 2020). This is the case of in intra-articular injections, for meniscus tears, the sealant for intervertebral disk tissue engineering and wounds (Agnol et al., 2019) (Abou-ElNour et al., 2020) (Tarafder et al., 2018) and for skin lesion in wound healing products (Mahmoud and Salama, 2016) (Prete et al., 2023). As medical devices for skin wound healing, there are several FDA-approved commercial products, in liquid form as fibrin glues, sealants, and fibrin patches, used as hemostatic agents and skin grafts.

**Fig. 9:**
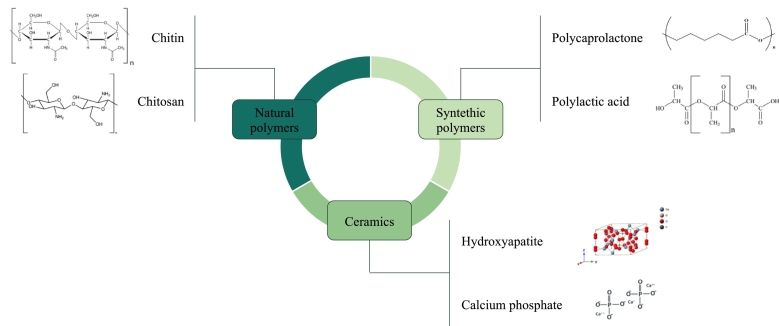
Classification of most used polymers.

A variety of biomaterials derived from natural and synthetic sources are widely used to manufacture scaffolds for tissue engineering and wound healing (Parupelli et al., 2023), as reported in Fig. 9. Among biomaterials, complex ones are also widely considered as bioceramics, hydrogels, polymer composites, nanocomposites (Biswal, 2021). Because of their superior mechanical properties, wettability, processability, and variable degradation rate, polymers are promising candidates in the design of scaffolds for tissue regeneration (Ambekar and Kandasubramanian, 2019) (Jafari et al., 2017).

### 6.1 Natural bioadhesives

The primary benefits of natural polymers, such as proteins and polysaccharides, over synthetic ones are their superior biocompatibility and quicker rate of complete degradation *in vivo*. This is a key point, since scaffolds should degrade slowly to be simultaneously replaced by the native tissue. Moreover, a synergism between scaffolds and cells is usually supported by natural polymers due to their intrinsic biological activity. Among natural polymers, chitosan, chitin fibrin, collagen and hyaluronate are mostly employed (Table 4).

**Table 4:** Most known and used bioadhesive materials and most common applications.

Bioadhesive materials	Chemical structure	Applications	Ref
Fibrin		Hemostatic agent, skin grafts (glues, sealants, patches)	(Sanz-Horta et al., 2023)
Chitosan		Mucoadhesive drug delivery systems, hemostatic agent, suture less surgery product, wound dressing	
Collagen		Tissue glues	(*Surgery*, 2001)
Cyanoacrylate		Alternative to suture during surgery, skin transplants, urgent wound care	(Petrie, 2014)

One of the most known natural bioadhesive is fibrin, which derives from fibrinogen and thrombin and is widely-used for scaffolds manufacturing due to its outstanding elasticity, deformability, biodegradability, and biocompatibility (Cassaro et al., 2019) (Sanz-Horta et al., 2023; Creste et al., 2020). Fibrin adhesives were introduced in the 1940s; among those, Beriplast®, TachoSil® and Veraseal™ are examples of commercially available fibrin-based surgical sealants, used for surgeries (Eberhard et al., 2006) (Montano et al., 2019) (Reichsöllner et al., 2023) although risk of allergy is not negligible, together with a high risk of contamination because of the presence of hemoderived products (Fernández-Vega-Cueto et al., 2022). Commercially available fibrin glues have been studied as alternatives to sutures for ophthalmic and periodonthal surgeries, proving their easier medical application and less post-operation pain than traditional sutures (Nachimuthu et al., 2025).

Alongside, collagen, which is a component of the ECM is the most frequent choice since it is characterized by excellent hemostatic properties, little antigenicity, minimal inflammatory response, and it favors cell migration and proliferation (Manjari et al., 2020) (Xiong et al., 2021). Collagen-based wound dressings, such as microspheres, hydrogels, electrospun membranes, porous scaffolds and films, are extremely promising for future wound care and are characterized by a good biocompatibility, remarked by both *in-vitro* and *in vivo* assays, although the poor mechanical properties represent a limitation (Zhang et al., 2012). Several *in-vivo* studies demonstrated that, after the implantation of collagen-based wound dressing, no immune-responses were recorded, demonstrating its suitability for biomedical applications (Zhang et al., 2012; Pamfil et al., 2017). Collagen is also successfully used in commercially available sealants products such as Floseal® and Costasis® (Andonegi et al., 2021) (Lee et al., 2019) (*Surgery*, 2001). Collagen-based sealants are relatively inexpensive and have a lower infection rate and diseases transmission risk compared to fibrin equivalents. Moreover, their adhesive strength can be enhanced by the addition of a crosslinker, expressing a higher tissue adhesion of eleven times in comparison with a fibrin sealant (Taguchi et al., 2004).

Other components of the ECM are glycosaminoglycans (GAGs), which are linear negatively-charged polysaccharides composed by repeating disaccharide units (one hexuronic acid and one amino sugar) linked by glycosidic bonds. They can undergo sulfation, acetylation, and epimerization to form complex structure; specifically, sulfated-GAGs can covalently interact with positively-charged proteins to form proteoglycans (Menezes et al., 2023) (Miller et al., 2014). On the contrary, hyaluronic acid is a non-sulfate GAGs, formed by repeating units of *N*-acetyl-D-glucosamine and glucuronic acid. Through its interactions with cell receptors, it also activates cells, controlling differentiation, motility, and inflammation and in particular adhesion. However, to boost cell attachment and engagement, chemical modification or crosslinking are required to form stronger structure that can support cell growth (Sodhi and Panitch, 2021). In this regard, a Schiff base cross-linking reaction was employed to create an *in situ* forming hydrogel based on chitosan and hyaluronic acid, able to enhance cellular responsiveness for abdominal tissue engineering (Aswathy et al., 2020). In addition, dopamine-conjugated maleic hyaluronic acid solution was photocrosslinked *in situ* to create a flexible hyaluronic acid hydrogel for hemostasis, expressing rapid gelation and strong adhesive properties. Specifically, the newly developed hydrogel proved to possess higher adhesion strength (179.9 ± 39.4 kPa) to tissues in comparison with the commercially available fibrin-base sealent (<40 kPa) (Fan et al., 2023). Hyaluronate esters (HYAFF) have also been developed to form 3D structure resistant to enzymatic degradation *in vivo* and able to sustain tissue reparation (Ruggeri et al., 2021).

In addition to collagen and hyaluronic acid, chitosan is another biomaterial characterized by high bioadhesive properties (Jing et al., 2017) (Ruprai et al., 2020) (Jung et al., 2021). In fact, chitosan, apart its mucoadhesive properties, possesses hemostatic properties, and biocompatibility (Liu et al., 2022b). Its polycationic behavior allows also to deeply interact not only with anionic moieties *via* charge-charge interaction but also with phospholipids in cell membrane *via* hydrophobic interactions (Jung et al., 2021). Alongside, chitosan based hydrogel as hemostatic agent proved strong adhesion to the lesion bed and improves wound healing process, reducing bleeding, avoiding infection, due to its antimicrobial properties, and accelerating healing (Song et al., 2024). The association of chitosan with other biopolymers has been considered to confer to the resulting structure increased mechanical properties as it was the case of collagen (Mahmoud and Salama, 2016). As example, collagen and chitosan hydrogels and sponge-like scaffolds have been successfully developed and characterized both *in-vitro* and *in-vivo*, showing promising results as bioadhesives in tissue engineering, as they favor cells homing, proliferation and ECM production (Mahmoud and Salama, 2016) (Yang et al., 2021) (Sánchez-Cid et al., 2022). HemCon Bandage Pro ChitoFlex® is a commercially available chitosan-based product, which can control bleeding caused by an extreme trauma; its efficacy was proved in preclinical studies and comparable to other hemostatic agents, providing 78 % and 100 % control of hemorrhage in two different studies respectively, which were conducted when there was a failure of gauze application (Devlin et al., 2011). However, when pressure is strong, the device tends to degrade over time and encourage fresh bleeding (Cassano et al., 2024).

### 6.2 Synthetic bioadhesives

Despite the advantages associated with their use, natural source derived biomaterials could have criticality in batch to batch reproducibility (Li et al., 2020). On the other side, synthetic polymers, such as polyesters, show defined and constant properties, as porosity, tensile strength, and degradability, which satisfy different needs (Biswal, 2021; Jafari et al., 2017).

Among synthetic bioadhesives for wound healing, cyanoacrylate has been the first surgical adhesives used in plastic surgery, skin transplants, and urgent wound care (Petter-Puchner et al., 2010) as some formulations have been FDA-approved as it is the cases of TRUFILL®, Dermabond® and Histoacryl® (Petrie, 2014) (Table 4). Because of their hazardous degradation products, and in particular formaldehyde, cyanoacrylates are only suitable for topical applications even if they have a high bonding strength and a little gluing time (Pascual et al., 2016). In fact, different studies demonstrated cyanoacrylated superior adhesive properties in comparison to fibrin adhesives. When applied on a pig colon suture, the pressure required to tear the suture line was investigated and it was found that a higher rupture force was required in case of cyanoacrylate-based adhesives (Alex and Re, 2025).

Progresses have been made in synthetic polymer-based adhesives and specifically, urethane-based adhesives are considered to be the most promising synthetic polymers (Uscátegui et al., 2017) (Ferreira et al., 2007). Semisynthetic polyurethane, having adhesive properties comparable to the commercially available bioadhesives, have been designed and synthetized using xylose monomers since xylose has 4 hydroxyl groups able to create hydrogen bonds between polymer chains and tissue and the adhesion propensity increases increasing the xylose substitution along the polyurethane chains (Balcioglu et al., 2016). Moreover the derivatization of polyurethane with acrylate allows to lead to UV crosslinked highly adhesive system (Balcioglu et al., 2021). A dual-components polyurethane biological adhesive composed by l-lysine diisocyanate (LDI), polyethylene glycol (PEG), and glycerol-synthesized polyurethane (PU) expressed higher adhesive properties on pig skin, showing a tensile modulus of 72 kPa, in comparison to fibrin glues (16 kPa). However, commercial cyanoacrylate glue goes over these values, with a tensile modulus of 135 kPa, proving it superior bioadhesiveness to pig skin and muscle (Ding et al., 2025).

## 7 Conclusions

Bioadhesive materials are key components in the biomedical and in particular, tissue engineering field, especially for the development of innovative tissue engineering devices and wound dressings. Bioadhesives possess a unique property since they can adhere to a biological surface (tissues, skin, mucosas) due to a variety of interactions, which make them interesting for the development of therapeutic platforms. Firstly, their ability to adhere to tissues was exploited to enhance drug delivery. Then, the use of those materials in regenerative medicine has attracted more interest, in particular as alternatives to sutures during surgery, hemostatic agent, sealants and treatments for wound and healing.

A crucial aspect in tissue engineering is related to the risk of an immunological reaction that significantly restricts the use of components (Vacanti et al., 2014; Mao and Mooney, 2015; Zou et al., 2022). In both surgical operations and trauma treatment, wound dressings with antimicrobial properties enable a favorable outcome lowering the risk of infection and speeding up the healing process (Agarwal et al., 2022). For this reason, materials and devices characterized by anti-inflammatory, self-healing and injectability features are particularly interesting (Duan et al., 2021). Innovative bioadhesives are able to activate biological pathways that can accelerate the healing process (Nerurkar et al., 2010; Guimarães et al., 2020). For example, innovative types of dressing are active adhesive dressings, which are able to apply contractile force to encourage active wound closure. These wound scaffolds are considered biomechanically active, as they can shrink and produce contractile forces once applied to the skin. Contractile forces are effectively transfer to the wound edges beneath, due to the highly adhesive features of the materials used to manufacture the dressings, supporting accelerated angiogenesis and re-epithelialization while also enhancing fibroblast populations with pro-regenerative characteristics (Blacklow et al., 2025).

Alongside with the increase of the use of bioadhesives, several techniques have been developed to investigate materials ability to adhere to biological substrate. One of the most known is tensile test and different biological substrates are employed. In fact, alongside the traditionally used pig skin and mucin, an artificial model, which should mimic skin, is employed, replacing the use of animal models.

Even though tensile test remains the most reliable technique; an international standard procedure to perform bioadhesive test has not been drafted yet, implicating a high variability in data collection and therefore quality lacks. To overcome this limitation, this review highlighted some parameters (withdrawal speed, contact time and preload force) that could be taken into account when thinking about a standard procedure. Moreover, work of adhesion is less impaired by those variables and could be more reliable in comparison to force of detachment. Mechanical tests have been considered since decades to characterize bioadhesion however they prove to give results related on the strength of adhesion and allow to elucidate the steps occurring in the formation of the adhesive joint. Moreover among the traditional techniques, rheological synergism contributes to give robust results affected by minimal variability about the actual formation of the bioadhesive joint. More recently developed techniques better allow to speculate on the mechanisms that drive the formation of the adhesion joint: this is the case of zeta potential for the charge-charge interaction or AFM for a specific moiety interaction. ITC is gaining attention of in this field because it allows not only to detect the formation of the bioadhesive joint but also to specifically measure the binding constant giving a quantitative evaluation of the affinity of the bioadhesive with a specific molecules of biological interest.

However each technique offers a different approach in the evaluation, based on the nature of the sample, its application and the different biological substrates used and the combination of the results offers the opportunity to go deep in the phenomenon and to understand which could be the formulative approach that better allow to obtain bioadhesion.

Although further studies are needed for standardized protocols that enable objective results, the employment of characterization using multiple techniques offer the possibility to obtain robust and reliable results. Along this, alternatives to animal tissue are in development to reduce the intrinsic variability of the results while obtaining insightful outcome.

## CRediT authorship contribution statement

**Marta Pollini:** Writing – review & editing, Writing – original draft, Methodology. **Eleonora Bianchi:** Visualization, Methodology. **Marco Ruggeri:** Visualization. **Barbara Vigani:** Visualization. **Silvia Rossi:** Resources. **Giuseppina Sandri:** Writing – review & editing, Funding acquisition, Conceptualization.

## Declaration of competing interest

The authors declare that they have no known competing financial interests or personal relationships that could have appeared to influence the work reported in this paper.

## Data Availability

Data will be made available on request.
